# Bilateral Choroidal Metastasis from Non-Small Cell Lung Cancer

**DOI:** 10.1155/2014/858265

**Published:** 2014-09-14

**Authors:** Tariq Namad, Jiang Wang, Annemarie Tilton, Nagla Abdel Karim

**Affiliations:** ^1^Division of Hematology/Oncology, Department of Medicine, University of Cincinnati, OH 45069, USA; ^2^Division of Pathology, Department of Medicine, University of Cincinnati, OH 45069, USA

## Abstract

Breast and lung cancers are the most common primary neoplasms to manifest with choroidal metastases. The incidence of choroidal metastases from metastatic lung cancer was reported to be 2–6.7%. We report a case of bilateral choroidal metastasis from non-small cell lung cancer. A 59-year-old Caucasian female patient, never a smoker, was diagnosed with stage IV lung adenocarcinoma metastatic to the pleura, bones, and the brain. Her initial scan of the chest showed innumerable soft tissue nodules and mediastinal adenopathy compatible with metastatic disease. Her initial brain MRI showed numerous small enhancing lesions consistent with extensive disease. Unfortunately, during her follow-up visits, she presented with bulge on her left eye. Simultaneously, her follow-up chest scan showed increase in the size of the lung nodules. She continued to have a reasonable performance status at that time, except for mild increase in her dyspnea. The choroidal metastases require a multidisciplinary care and should be among the differential patients with malignancy who present with ocular symptoms.

## 1. Background

Breast and lung cancers are the most common primary neoplasms to manifest with choroidal metastases [1]. The incidence of choroidal metastases from metastatic lung cancer was reported to be 2–6.7% in clinical trials, where it was 0–9.7% in metastatic breast cancer. However, it was shown to be as high as 30% in several reports [2–5]. The choroidal metastases are often asymptotic and, thus, their diagnosis remains challenging. They are generally associated with an advanced disease. Their frequency might be underestimated.

## 2. Case Presentation

**Figure 1:**
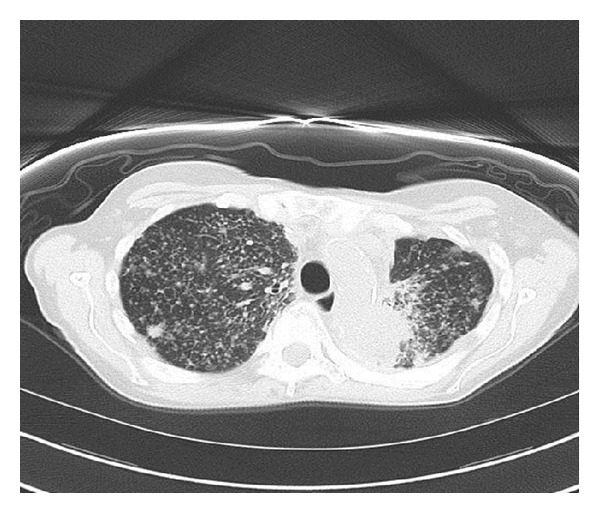
Chest CT without contrast: pulmonary nodules of varying sizes persisting throughout the lung zones, with the largest shown here in the right upper lobe.

**Figure 2:**
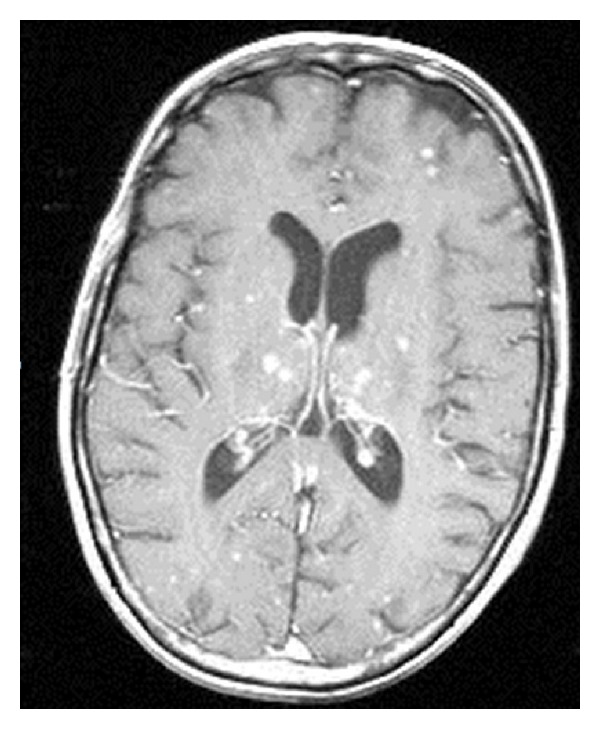
MRI Brain with and without contrast: T1 axial postcontrast image showing diffuse nodule enhancement, most pronounced in the thalamic regions and basal ganglia.

We report a case of a 59-year-old Caucasian female patient, never a smoker, with a remote history of Hodgkin's lymphoma from which she was cured. She has received a total nodal irradiation around 30 years prior to her lung cancer diagnosis. Unfortunately, she was diagnosed with stage IV lung adenocarcinoma metastatic to the pleura, bones, and the brain, where the pleural biopsy confirmed the diagnosis with EGFR exon 19 and exon 21 negative mutations and EML4-ALK gene rearrangement was also negative. Her initial scan of the chest showed innumerable soft tissue nodules of varying sizes and mediastinal adenopathy compatible with metastatic disease (Figure 1). Her initial brain MRI showed numerous small enhancing lesions throughout the posterior fossa and cerebral hemispheres bilaterally consistent with extensive disease (Figure 2) along with cervical spine lesions.

She underwent pleuroscopy and talc pleurodesis for the malignant pleural effusion with approximately 2 liters of fluid removed and then palliative radiation therapy for spine lesions to 6th and 7th cervical vertebraes for her back pain, but her brain lesions were too small and she was asymptomatic. This was followed by systemic chemotherapy with carboplatin and pemetrexed for four cycles followed by maintenance pemetrexed. She also received zoledronic acid for her skeletal metastases. She remained on the pemetrexed maintenance for 14 cycles, which she tolerated very well, and had minimal response versus stable disease. Unfortunately, during her follow-up visits, she presented with bulge on her left eye. Simultaneously, her follow-up chest scan showed increase in the size of the lung nodules. She continued to have a reasonable performance status at that time, except for mild increase in her dyspnea, with normal oxygen saturation.

**Figure 3:**
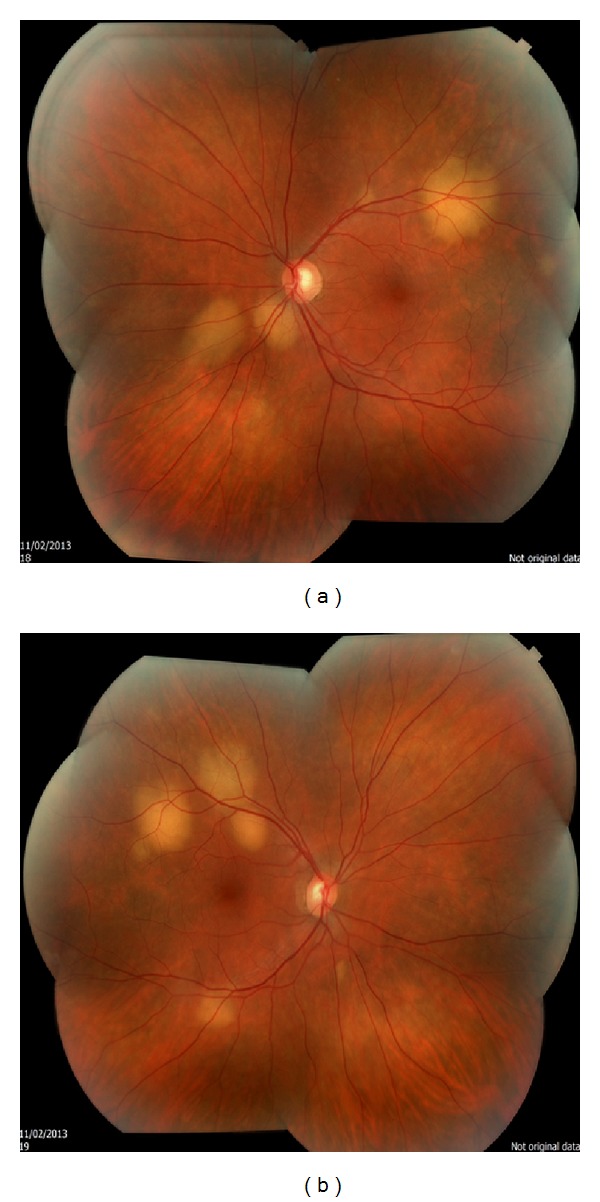
A fundoscopic examination revealed the presence of multiple choroidal masses in both eyes.

Ophthalmology consultation was obtained for her new left eye bulging and the plan was to initiate a second line of systemic therapy then. However, ophthalmologic exam revealed bilateral multiple cream-colored choroidal infiltrative tumors consistent with metastatic tumors (Figures 3(a) and 3(b)). The ultrasound and fluorescein angiography techniques have not been utilized. A follow-up brain MRI which was also obtained revealed progressive brain metastatic lesions.

She underwent whole brain and posterior eyes radiation, 300 centigrays (cGy) per fraction with a 3000 cGy total dose, but unfortunately her condition deteriorated very shortly afterwards and she was enrolled to hospice and could not receive second line systemic therapy due to rapid decline in performance status. She died few weeks after the diagnosis and treatment of the choroidal and brain metastases.

## 3. Discussion

Intraocular metastasis is considered the most common malignancy of the eye [2]. The highly vascular uveal tract is the most common part of the eye involved by metastases. Within the uvea, the choroid (88%) is the most commonly affected site followed by the iris (9%) and ciliary body (2%) [6].

Among women, the primary sites for choroidal metastasis are from the breast, lung, unknown primary, gastrointestinal and pancreas, cutaneous melanoma, and other rare sources. Among men, however, the primary sites are the lung, cancer of unknown primary, gastrointestinal and pancreas, prostate, kidney, cutaneous melanoma, and other rare sources [1, 7, 8]. Kreusel et al. [4] studied 84 patients with primary lung cancer, where the prevalence of the choroidal metastases was estimated to be 7.1%. This was mostly associated with at least two other metastatic sites. Others reported the presence of choroidal metastases as high as 9% [9]. Mewis and Young [10] found a frequency of 7%, for choroid metastasis from breast cancer.

On the other hand, Barak et al. [11] in a study from Tel Aviv Sourasky Medical Center screened 77 patients with advanced breast cancer and 92 patients with advanced lung cancer; they noticed an incidence lower than that described previously with no cases of choroidal metastases for the breast cancer patients and 2 cases for the lung cancer patients. Moreover, Fenton et al. [12] found no choroidal metastasis among 68 screened patients with advanced metastatic disease of the breast.

The metastasis could be unilateral or bilateral. Shields et al. [1, 13] followed 420 consecutive patients with uveal metastases where tumors were bilateral in 23.80% and unilateral in 76.20%. Nicolò et al. [13], on a clinicopathologic study of 227 cases for carcinoma metastatic to the eye and orbit, found more than 4.4% of bilateral cases. Mewis and Young in analysis of 67 patients with breast carcinoma metastatic to the choroid [14] noted a 31% incidence of bilaterality. In both the Ferry/Font and Mewis/Young studies, the incidence of subsequent bilaterality was notable as 17.6% and 15%, respectively. There seems to be no predilection for metastasis to preferentially affecting the right or left eye [2, 13, 15].

Choroidal metastases are usually associated with other metastatic sites in more than 70% of the cases [15–17]. They are seldom the solitary site of metastases. Generally, they are often asymptomatic but can present with blurry vision, pain, photopsia, red eye, floaters, and a decrease or even loss of vision in the absence of macular invasion [4, 8, 17–19]. In the majority of cases, the diagnosis is established by slit-lamp exam. It usually reveals the yellow-white elevated lesion in the choroid with possible serous retinal detachment. The choroidal metastases are generally located on the posterior part of the equator that is richly vascularized [20].

The diagnosis might be challenging in cases where the primary malignancy is not yet established or the choroidal metastasis is the first sign. Choroidal metastases could mimic other conditions as choroidal melanoma, choroidal osteoma, choroidal hemangioma, choroidal neovascularization with disciform scar, posterior scleritis, and other rare lesions [21].

The use of ultrasound aids in the diagnosis. It usually shows an echogenic subretinal mass with diffuse, ill-defined borders as well as the fluorescein angiography in which these lesions are usually fluorescent in the early phases of the study and become progressively hyperfluorescent in the late phases [22]. Mostly no biopsy is needed unless extensive staging work-up had failed to reveal the primary or other metastatic sites [23].

The computed tomography, fine needle aspiration, or wedge biopsy can be considered as a part of diagnostic panel. The brain MRI is useful before initiation of radiotherapy to assist in treatment planning. It is reported that 22% of patients diagnosed with choroidal metastasis had a concurrent diagnosis of central nervous system metastasis [24, 25].

The treatment modalities for choroidal metastases are external beam radiotherapy, plaque radiotherapy, surgical resection, or transpupillary thermotherapy. The intravitreal chemotherapy remains investigational [21].

### 3.1. Radiation Therapy

The radiation therapy includes whole choroid irradiation, curietherapy by plates of iodine 125 (I-125), and the stereotactic radiotherapy. Choroid irradiation with a dose of 30 Gy in 10 sessions on the posterior segment of the affected eye allows protecting the vision and avoiding the enucleation. The response rate is almost 80% of the cases with preservation of the eyeball in almost 100% of the cases [17, 20, 26, 27].

The external beam radiation seems to be the recommended modality for the intraocular metastases [24, 28]; 43% of the patients maintain their initial vision and 26% have an improvement of their vision. The phototherapy seems to be another interesting approach, where in a recent retrospective study of 63 patients (76 eyes) with a choroidal metastasis a complete regression of the lesions was observed in 94% of the cases in five months without relapse, a control of the detachment of retina in 82% in 3, 8 months, and an improvement or a stabilization of the visual acuteness in 47% of the cases [20, 29].

### 3.2. Systemic Therapy

The use of chemotherapy alone for choroidal metastases is not widely reported. Some authors showed an improvement of the visual acuteness under chemotherapy in the literature and they concluded that chemotherapy alone can be used as an effective mode of treatment in patients who have primary tumors that are chemosensitive [21, 30]. The assessment of response to their treatment can be assessed by fundoscopy, by B-scan ultrasound, and clinically by the improvement in visual acuity [21, 31]. Letson et al. [32] who treated 6 patients with breast cancer and choroidal metastasis concluded that systemic chemotherapy is as effective as radiotherapy as all 6 patients treated with systemic chemotherapy alone showed regression of their choroidal metastasis. Barry et al. [20] described four cases with choroidal metastases from a non-small cell lung cancer and showed that the systemic therapy (with multiple chemotherapy's agents) does not seem to be effective.

In another report by Shimomura et al. [33] from Japan, a female patient who presented with choroidal metastasis from a non-small lung cancer with epidermal growth factor receptor (EGFR) mutation was treated with Gefitinib, a tyrosine kinase inhibitor (TKi). They concluded the effective use of EGFR-TKis for choroidal metastasis of NSCLC with an EGFR mutation, to prevent or at least delay irreversible visual loss, and then they suggested that the EGFR-TKi should be initiated earlier and should be continued as long as possible during treatment.

The intravitreal therapy could be an effective therapy to the choroid metastases especially if it is combined with systemic therapy. A prior report of a female patient from South Korea treated with intravitreal bevacizumab (a Vascular endothelial growth factor (VEGF) inhibitor and an oral tyosine kinase; erlotinib combination therapy for choroidal metastases secondary to NSCLC. Best corrected visual acuity (BCVA), fluorescein angiography (FA), optical coherence tomography (OCT), and B-scan ultrasonography were compared during the 4-month treatment period. Four weeks after the third injection of bevacizumab (2.5 mg), the BCVA had improved to 20/40 from 20/200 and the 2 subretinal masses had completely disappeared. FA demonstrated only a retinal pigment epithelial (RPE) window defect with minimal to no leakage. In the B-scan ultrasonography and OCT, no further mass-like lesion was detected. The retina and RPE layer were flattened. combining intravitreal bevacizumab and oral erlotinib could be another treatment option for patients with choroidal metastasis of NSCLC [34].

## 4. Conclusion

It appears that choroidal metastases are rare and tend to occur in the advanced stages of cancer, where the mean survival is not expected to be more than 6 months [25, 35]. The incidence is still controversial, but it seems that it might increase in the presence of maintenance therapies and longer survival of patients with lung cancer. In the past, choroidal metastasis was treated with radiotherapy alone or in combination with chemotherapy. More recently, targeted therapy could be effective, if the primary cancer seems to have a good response to this treatments modality. Local therapy or intravitreal therapy is another promising option.

Choroidal metastases require multidisciplinary care and should be among the differential patients with malignancy who present with ocular symptoms. Ophthalmology referral should be considered in these cases. Whether systemic chemotherapy concomitant with local radiation therapy or prior to radiation therapy would have been used to improve the outcome remains a challenging question. More studies to explore therapeutic options for patients with advanced NSCLC and choroidal metastases are warranted.
